# The contribution of visceral fat to improved insulin signaling in Ames dwarf mice

**DOI:** 10.1111/acel.12201

**Published:** 2014-02-12

**Authors:** Vinal Menon, Xu Zhi, Tanvir Hossain, Andrzej Bartke, Adam Spong, Adam Gesing, Michal M Masternak

**Affiliations:** 1 College of Medicine, Burnett School of Biomedical Sciences, University of Central FloridaOrlando, FL, 32827, USA; 2 Department of Obstetrics and Gynecology, Peking University Third Hospital, Center of Reproductive MedicineBeijing, 100191, China; 3 Department of Internal Medicine, Southern Illinois University School of MedicineSpringfield, IL, 62794, USA; 4 Department of Oncological Endocrinology, Chair of Oncological Endocrinology, Medical University of LodzLodz, Poland; 5 Institute of Human Genetics, Polish Academy of SciencesPoznań, Poland

**Keywords:** adiponectin, adipose tissue, Ames dwarf, insulin, obesity

## Abstract

Ames dwarf (*Prop1*^*d*f^, df/df) mice are characterized by growth hormone (GH), prolactin, and thyrotropin deficiency, remarkable extension of longevity and increased insulin sensitivity with low levels of fasting insulin and glucose. Plasma levels of anti-inflammatory adiponectin are increased in df/df mice, while pro-inflammatory IL-6 is decreased in plasma and epididymal fat. This represents an important shift in the balance between pro- and anti-inflammatory adipokines in adipose tissue, which was not exposed to GH signals during development or adult life. To determine the role of adipose tissue in the control of insulin signaling in these long-living mutants, we examined the effects of surgical removal of visceral (epididymal and perinephric) adipose tissue. Comparison of the results obtained in df/df mice and their normal (N) siblings indicated different effects of visceral fat removal (VFR) on insulin sensitivity and glucose tolerance. The analysis of the expression of genes related to insulin signaling indicated that VFR improved insulin action in skeletal muscle in N mice. Interestingly, this surgical intervention did not improve insulin signaling in df/df mice skeletal muscle but caused suppression of the signal in subcutaneous fat. We conclude that altered profile of adipokines secreted by visceral fat of Ames dwarf mice may act as a key contributor to increased insulin sensitivity and extended longevity of these animals.

## Introduction

Ames dwarf (df/df) mice, discovered at Iowa State University, are homozygous for a spontaneous mutation in the *prophet of pituitary factor-1* (*prop1*) gene, which codes for a transcription factor that is responsible for pituitary development. Due to the loss-of-function mutation, df/df mice are devoid of three cell types in the anterior pituitary gland – somatotrophs, lactotrophs, and thyrotrophs – leading to hormonal deficiencies of growth hormone (GH), prolactin (PRL), and thyroid-stimulating hormone (TSH). Lack of GH leads to these mutants having extremely low levels of insulin-like growth factor-1 (IGF-1) in circulation and a dwarf phenotype. These mice tend to get obese as they age, but yet live significantly longer compared with their normal (N) siblings. Ames dwarf mice not only exhibit an increase in longevity, living 40–60% longer relative to N mice, but also an enhanced healthspan, maintaining their youthful appearance with age. These mutants show no signs of diabetes; they are extremely insulin sensitive with low levels of glucose and insulin in circulation (Brown-Borg *et al*., 1996; Bartke *et al*., 2001, 2008). Ames dwarf mice have improved cognitive function and show no age-related decline in memory (Kinney *et al*., 2001) and are less prone to cancer (Ikeno *et al*., 2003).

Surgically removing visceral fat (VF) depots have led to an improvement in glucose tolerance and insulin sensitivity in laboratory animals (Barzilai *et al*., 1999; Kim *et al*., 1999; Gabriely *et al*., 2002; Lottati *et al*., 2009), supporting the idea that VF represents ‘bad’ fat and is associated with several disease conditions including diabetes. On the other hand, the fact that subcutaneous (SQ) fat is ‘good’ body fat was strengthened by fat transplant experiments carried out by Tran and his colleagues, where beneficial effects on insulin sensitivity and glucose tolerance were achieved by transplanting SQ fat into the visceral cavity (Tran *et al*., 2008).

It is well documented that adipose tissue does not just function passively to store fat, but is an endocrine organ secreting several biologically active peptides known as adipokines which include adiponectin, TNFα, and IL-6 (Coelho *et al*., 2013). The level of anti-inflammatory and antidiabetic adiponectin decreases with an increase in obesity (Hu *et al*., 1996; Kern *et al*., 2003; El-Wakkad *et al*., 2013), while that of pro-inflammatory adipokines TNFα and IL-6 increases with the accumulation of VF (Kern *et al*., 2001). This shift in the secretion of adipokines under obese conditions leads to a chronic state of low-grade systemic inflammation.

Glucose and insulin/IGF-1 – like pathways in yeast, worms, and flies – lead to growth as well as aging of these organisms. Mutations that suppress or perturb these pathways lead to an increase in longevity (Longo & Finch, 2003). These ‘longevity’ pathways are also conserved in mammals. There are several types of mice with spontaneous mutations or targeted disruptions of genes related to the GH/IGF-1 (somatotropic axis), including the growth hormone receptor/binding protein knock out (GHRKO) mouse, that have extended longevity (Bartke, 2005). The GHRKO mouse was developed as an animal model for Laron syndrome that affects humans (Zhou *et al*., 1997). Human subjects with this disorder have a mutation in the GHR gene leading to a dwarf phenotype (Guevara-Aguirre *et al*., 2011). Like the animal model, Laron dwarf humans have central obesity, low levels of IGF-1, and high levels of GH in circulation and due to the absence of functional GHRs, are resistant to the actions of GH. Also, these subjects seem to be protected from cancer and diabetes (Zhou *et al*., 1997; Guevara-Aguirre *et al*., 2011). Thus, also in humans, disruption of the GH/IGF-1 axis could potentially extend lifespan. In contrast, transgenic mice overexpressing GH have high circulating IGF-1 and a ‘giant’ phenotype and are characterized by a decreased insulin sensitivity as well as reduced longevity (Bartke *et al*., 2002). Humans with a pathological excess of GH (acromegaly) are also insulin resistant (Wasada *et al*., 1997) and have reduced life expectancy. Moreover, an early-life administration of GH to long-living df/df mice leads to reduced insulin sensitivity (Masternak *et al*., 2010) and a decrease in longevity (Panici *et al*., 2010). Thus, GH seems to play an important role in modulating insulin sensitivity and longevity. As mentioned earlier, df/df mice live significantly longer compared with N mice. However, when these mutants are subjected to calorie restriction (CR), there is a further increase in longevity (Masternak *et al*., 2009). This extension of longevity is reflected in improved insulin sensitivity as measured by an insulin tolerance test (ITT); df/df mice are very insulin sensitive compared with N mice, and there is a further increase in insulin sensitivity in these mutants when calorie intake is reduced (Masternak *et al*., 2009). Not only are the changes in the levels of proapoptotic factors and key regulators of mitochondrial biogenesis not further improved by calorie restriction in GHRKOs (Gesing *et al*., 2013); interestingly, there is no further extension of longevity or an increase in insulin sensitivity when these long-living GHRKO mice are subjected to this same dietary intervention (Masternak *et al*., 2009). Thus, there is a probable link between insulin sensitivity and longevity.

The fact that df/df mice are inclined to get obese as they age but yet maintain high adiponectin levels in circulation (Bartke *et al*., 2008) and live longer and healthier lives compared with N mice leads us to hypothesize that VF in these mutants, developed in the absence of GH signaling, contributes to the enhanced insulin sensitivity of df/df mice, presumably by secreting low levels of pro-inflammatory IL-6 and increased levels of anti-inflammatory adiponectin.

## Results

### Effect of VFR on glucose tolerance and insulin sensitivity

**Figure 1:**
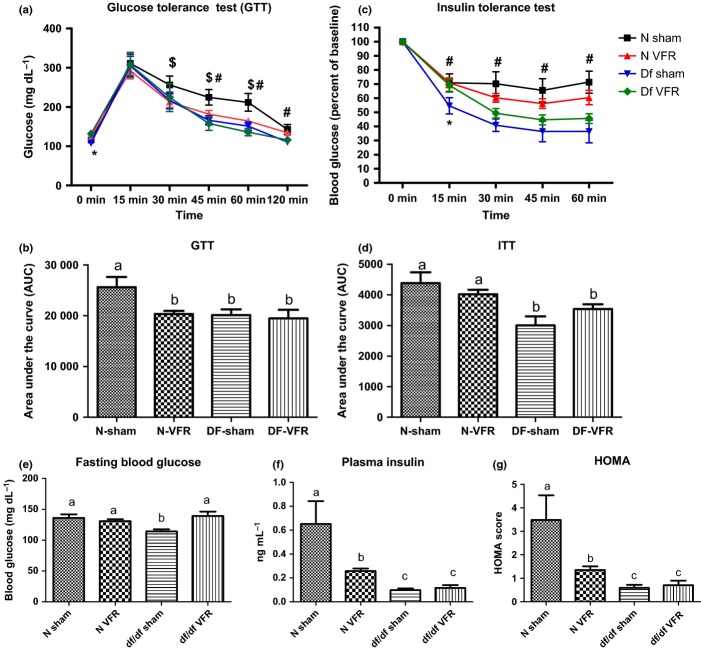
Effect of VFR on glucose tolerance and whole-body insulin sensitivity. (A, B, C, D) Results of glucose tolerance test (GTT) and insulin tolerance test (ITT). (E) Effect of VFR on fasting blood glucose levels. (F) Effect of VFR on plasma insulin levels. (G) Insulin sensitivity measured by HOMA analysis. Different letters represent statistical significance (*P* < 0.05). $, *, and # represent statistical significance between N sham and N VFR, df/df sham and df/df VFR, N Sham and df/df sham, respectively.

As revealed by the glucose tolerance test (GTT), only N mice that were subjected to VFR (N-VFR) showed an improvement in glucose tolerance with no difference in Ames dwarf mice after VFR (df/df VFR) (Fig. 1A, B). There was a significant difference in blood glucose levels between N mice in the sham-operated and VFR groups at the following time points: 30 min (*P* = 0.0436), 45 min (*P* = 0.0274), and 60 min (*P* = 0.0249). The difference in blood glucose between N-sham and df/df-sham mice was significant only at 45 min (*P* = 0.0079), 60 min (*P* = 0.0104), and 120 min (*P* = 0.0100) time points. Interestingly, at time 0 min, mice in the df/df-sham group had a significantly lower blood glucose level than mice in the df/df-VFR group (*P* = 0.0028) (Fig. 1A). Additional analysis of area under the curve (AUC) indicated improved glucose tolerance in N VFR, df/df sham, and df/df VFR when compared to N-sham mice (*P* = 0.0086, *P* = 0.0125, and *P* = 0.0122, respectively) (Fig. 1B), while glucose tolerance in df/df VFR did not differ from values measured in df/df-sham animals.

As expected, df/df-sham mice were very insulin sensitive compared with N-sham mice. During the insulin tolerance test (ITT), blood glucose levels (expressed as percent of baseline) differed between df/df and N animals at 15 min (*P* = 0.0381), 30 min (*P* = 0.0038), 45 min (*P* = 0.0090), and 60 min (*P* = 0.0034). The effect of VFR on insulin sensitivity in df/df mice was statistically significant only at the 15-min time point (*P* = 0.0297). There was significant genotype/intervention interaction for GTT and ITT as determined by repeated-measures ANOVA test (*P* = 0.0310 and *P* = 0.0006 respectively) (Fig. 1C). Area under the curve analysis also indicated increased insulin sensitivity of df/df-sham and df/df-VFR mice compared with N-sham animals (*P* = 0.0041 and *P* = 0.0122, respectively). There was also a trend for decreased insulin sensitivity in df/df VFR compared with df/df-sham mutants which did not reach statistical significance (*P* = 0.0615) (Fig. 1D).

Two-way ANOVA analysis did not reveal significant effects of either genotype or intervention (VFR) on fasting blood glucose levels (FBG); however, there was significant genotype and intervention interaction (*P* < 0.0056). Following two-way ANOVA, *t*-test indicated significantly lower levels of FBG in df/df mice as compared to N mice (*P* = 0.0014). Although VFR did not have any effect on FBG of N mice, this surgical intervention led to a significant increase in FBG of df/df mice (*P* = 0.0020) (Fig. 1E).

There was a significant effect of genotype on insulin levels as revealed by two-way ANOVA (*P* < 0.0024). Further analysis indicated that plasma insulin levels were significantly lower in the mutants compared with N mice (*P* = 0.0125). Surgically removing VF depots led to a significant decrease in circulating insulin levels in N mice only (*P* = 0.0233) with no change in df/df mice (Fig. 1F). Two-way ANOVA of the homeostatic model assessment (HOMA) score indicated significant genotype effect (*P* < 0.0058). Further analysis revealed that df/df-sham mice were markedly more insulin sensitive than the N-sham mice (*P* = 0.0153). Also, in parallel with decreased plasma insulin levels, VFR significantly improved insulin sensitivity only in N mice (*P* = 0.0306) (Fig. 1G).

### Effect of VFR on insulin signaling in skeletal muscle

**Figure 2:**
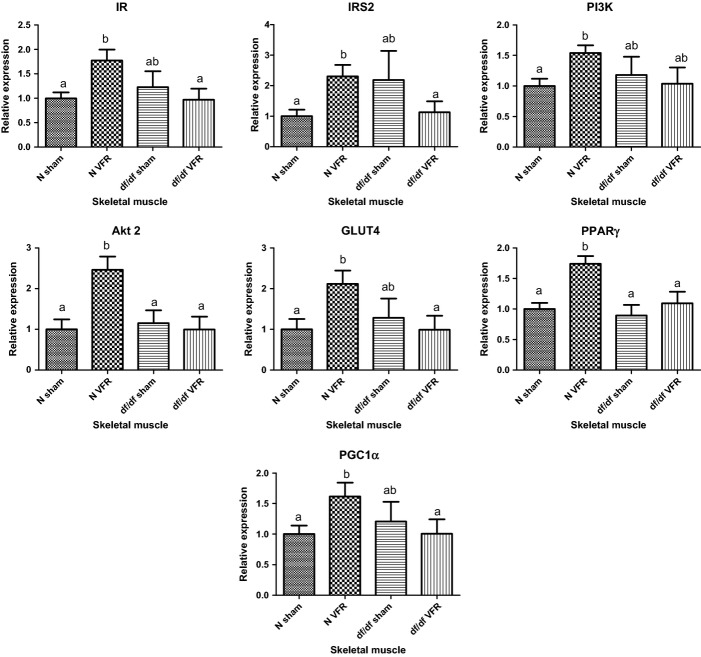
Differential gene expression upon VFR in skeletal muscle of N and df/df mice. Different letters represent statistical significance (*P* < 0.05).

Two-way ANOVA analysis of the expression levels of genes involved in insulin signaling pathway in skeletal muscle revealed significant interaction between genotype and intervention for mRNA levels of insulin receptor (IR), insulin receptor substrate-2 (IRS-2), and Akt2 (*P* < 0.0223, *P* < 0.03 and *P* < 0.0222 respectively) and a similar trend for glucose transporter 4 (GLUT4) (*P* < 0.056). Moreover, expression of peroxisome proliferator-activated receptor gamma (PPARγ) was significantly affected by both genotype and intervention (*P* < 0.0196 and *P* < 0.0043, respectively) without any significant level for interaction between genotype and intervention (*P* < 0.0867). Further analysis of transcript levels of IR, IRS2, phosphoinositide 3-kinase (PI3K), Akt2, GLUT4, PPARγ, and peroxisome proliferator-activated receptor gamma coactivator 1-alpha (PGC1α) showed significant increase in skeletal muscle of N-VFR mice compared with N-sham group (*P* = 0.0054, *P* = 0.0046, *P* = 0.0032, *P* = 0.0014, *P* = 0.0085, *P* = 0.0002, and *P* = 0.0187, respectively) with no alterations in df/df-VFR when compared to df/df-sham mutants (Fig. 2).

### Effect of VFR on the expression of insulin signaling genes in SQ fat

**Figure 3:**
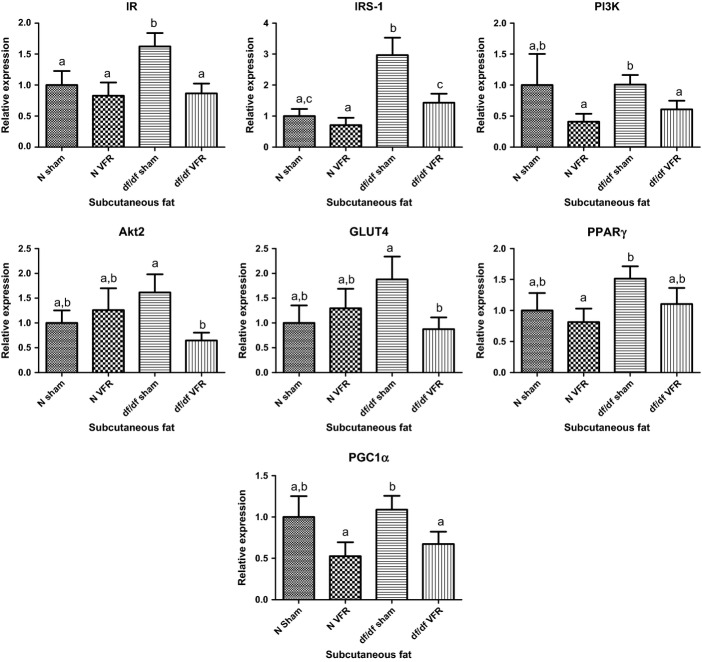
Effect of VFR on gene expression in subcutaneous fat. Different letters represent statistical significance (*P* < 0.05).

Two-way ANOVA analysis of real-time PCR data indicated significant effects of VFR on the gene expression levels of IR, IRS1, and PGC1α (*P* < 0.03; *P* < 0.01; and *P* < 0.028, respectively). This analysis also indicated significant genotype effect on the expression of IRS1 in SQ fat (*P* < 0.0003). Additional analysis following our initial findings by two-way ANOVA indicated a decrease in the expression of IR, IRS-1, PI3K, Akt2, GLUT4, and PGC1α genes in SQ fat after VFR in df/df mice only (*P* = 0.0057, *P* = 0.0115, *P* = 0.0362, *P* = 0.0098, *P* = 0.0326, and *P* = 0.0428, respectively) without any alterations in N animals subjected to the same surgical procedure. Moreover, the mRNA expression of IR and IRS-1 was higher in df/df-sham mice compared with N-sham mice (*P* = 0.0336 and *P* = 0.0019 respectively) (Fig. 3).

### Differential gene expression and secretory profile of visceral adipose tissue and circulating adipokine levels in df/df vs. N mice

**Figure 4:**
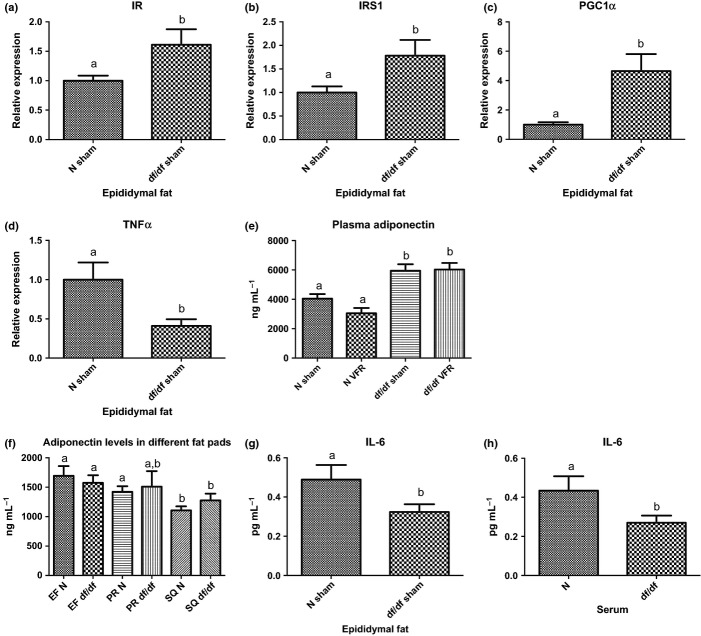
Gene expression levels in epididymal fat and adipokines levels in circulation. (A, B, C) Expression of genes promoting insulin sensitivity in epididymal fat. (D) Expression of TNFα transcript in epididymal fat. (E, F) Adiponectin levels in circulation and in different fat pads. (G, H) IL-6 protein levels in EF and serum. Different letters represent statistical significance (*P* < 0.05)

In this experiment, we used epididymal fat (EF) as a main source of visceral fat, collected from animals in the sham-operated group. The relative expression levels of IR, IRS-1, and PGC1α were significantly higher in EF from df/df mice when compared to the same fat depot from N mice (*P* = 0.0209, *P* = 0.0221, and *P* = 0.0033 respectively) (Fig. 4A, B, C). In contrast, the RNA expression level of pro-inflammatory TNFα was down-regulated in EF of df/df mice relative to EF from N mice (*P* < 0.0114) (Fig. 4D). As documented previously in the literature, Ames dwarf mice had significantly higher levels of adiponectin in circulation (*P* = 0.0031), although VFR did not alter circulating levels of this adipokine in either group of mice (Fig. 4E). More direct analysis of adiponectin at the protein level by ELISA in epididymal (EF), perirenal (PR), and subcutaneous (SQ) fat pads, dissected from a second cohort of male mice as mentioned in materials and methods, did not show any differences between df/df and N mice (~1 year old) (Fig. 4F).

However, EF and PR fat had higher adiponectin protein levels as compared to SQ fat in N mice (*P* = 0.0038 and *P* = 0.0139 respectively). Also, the EF depot in df/df mice had higher levels of adiponectin protein compared with SQ fat of these mutants (*P* = 0.0489) (Fig. 4F). Bio-Plex analysis revealed significantly lower protein levels of IL-6 in EF of df/df mice relative to the N mice (*P* = 0.0308) (Fig. 4G). Parallel to decreased protein expression of IL-6 in EF of df/df mice, these mutants also had low levels of this pro-inflammatory adipokine in circulation relative to control mice (~1 year old) (*P* = 0.0495) (Fig. 4H).

## Discussion

Ames dwarf mice tend to get obese as they age; however, they not only live longer, but also show an increase in healthspan, with delayed signs of age-related diseases including diabetes. Thus, we believe that the VF depots in these mutants are functionally distinct from the same fat depots in N mice and are beneficial, rather than detrimental, to the overall health of these mutant mice.

Growth hormone-deficient df/df mice share many physiological and endocrine characteristics with GH-resistant GHRKO mice. Surgical removal of VF depots in GHRKO mice strengthened the idea that these fat depots, developed in the absence of GH signaling, have beneficial effects (Masternak *et al*., 2012). This intervention improved insulin sensitivity only in control mice, with opposite effects in GHRKO mutants (Masternak *et al*., 2012).

As expected, df/df mice had improved glucose tolerance and insulin sensitivity, as indicated by GTT and ITT, compared with N mice. However, the surgical removal of VF depots improved glucose tolerance only in the control mice. There was no significant effect of VFR on insulin sensitivity on either group of mice, as measured by ITT although numerically insulin sensitivity was increased in N mice and reduced in the dwarfs. The HOMA score, which is a measure of insulin sensitivity, calculated by the formula (insulin × glucose)/22.5, with lower values pointing to higher insulin sensitivity, indicated that VFR improved insulin sensitivity only in N mice, with a parallel decrease in plasma insulin levels in these controls. Also, in conjunction with ITT results, HOMA scores revealed that df/df mice were much more insulin sensitive compared with N mice. One possible explanation for the lack of a significant effect of VFR on insulin sensitivity measured by ITT is that this test is accompanied by a high risk of inducing hypoglycemic conditions in the test mice, which induces endogenous glucose production and glycogenolysis to maintain euglycemic levels, thus hampering test results. Given the small body size of these mutant dwarfs, it is technically not feasible to perform the more sensitive hyperinsulinemic-euglycemic clamp study as a measure of insulin sensitivity.

Even though there was a decrease in plasma insulin levels of N mice that were subjected to VFR, there was no change in blood glucose levels of this group of mice. This indicates that the decreased levels of insulin upon VFR are sufficient to maintain euglycemic levels in N mice in the VFR group. However, this surgical intervention led to an increase in blood glucose in df/df mice with no change in plasma insulin levels. Thus, surgical removal of VF depots in N mice improved whole-body insulin sensitivity, supporting previous data, as mentioned before, that show that these fat pads represent ‘bad’ body fat; but in df/df mice, removal of the same fat depots negatively affects blood glucose levels, suggesting that VF in the mutants plays a positive role in maintaining whole-body insulin sensitivity.

Skeletal muscle and adipose tissue are two of the most insulin-responsive organs. Surgically removing VF depots led to an up-regulation of several genes involved in the insulin signaling pathway in the skeletal muscle of N mice; in turn, this procedure led to a decrease in the expression of these genes in SQ fat of df/df mice. This decreased expression of the insulin signaling genes, including the glucose transporter GLUT4, in SQ fat and the absence of functionally beneficial VF (due to VFR) in the df/df mice could probably lead to decrease glucose disposal and a corresponding increase in blood glucose.

The up-regulation of some of the basic players involved in insulin signaling in skeletal muscle of N, but not df/df mice, subjected to VFR indicates that only normal mice benefit from this surgical intervention. The increased mRNA expression of insulin receptor (IR), insulin receptor substrate-2 (IRS-2), phosphoinositide 3-kinase (PI3K), Akt 2, and GLUT4 in the skeletal muscle of the N mice with visceral fat removed probably contributes to the increased insulin sensitivity seen in these animals which corresponds with previous findings in normal controls from a different strain (Masternak *et al*., 2012). However, to fully understand the effects of insulin action in skeletal muscle and other insulin target organs, further functional analysis of the levels of phosphoproteins corresponding to the analyzed genes needs to be performed in future as was carried out in our studies of VFR in GHRKO mice (Masternak *et al*., 2012).

Peroxisome proliferator-activated receptors (PPARs) are ligand-dependent transcription factors activated by different ligands, such as fatty acids, and are targets of the antidiabetic class of drugs known as thiazolidinediones (TZDs). These nuclear receptors, once activated, form heterodimers with retinoid X receptors (RXRs) and bind to peroxisome proliferator response elements (PPREs) in the enhancers of target genes leading to activation of transcription of these genes (Masternak & Bartke, 2007). PPARγ is highly expressed in white adipose tissue; however, several insulin-sensitive tissues also express this transcription factor. Skeletal muscle PPARγ increases glucose uptake and utilization (Auwerx *et al*., 2003), and optimum expression of this nuclear receptor seems to play an important role in maintaining whole-body insulin sensitivity. Muscle-specific PPARγ knockout mice exhibit an increase in adiposity and develop insulin resistance, but are still responsive to TZDs (Norris *et al*., 2003). On the other hand, CR – an intervention that improves whole-body insulin sensitivity – led to a decrease in the expression of PPARγ in skeletal muscle of GHRKO mice (Masternak & Bartke, 2007). In the present study, VFR led to increased transcript levels of PPARγ, as well as its coactivator – PGC1α in skeletal muscle of N mice only; thus, it probably contributes to increased insulin sensitivity seen in this group of mice.

The effect of VFR on SQ fat was studied by qPCR analyses of the expression of genes involved in insulin signaling in this fat depot. The decrease in mRNA levels of IR, IRS-1, PI3K, Akt 2, and GLUT4 in SQ fat of df/df mice in the VFR group may, in part, contribute to decreased insulin signaling in this fat depot, and a corresponding increase in blood glucose seen in these long-living mice. Mice with adipose-specific knockout of PGC1α are characterized by insulin resistance when fed a high-fat diet (Kleiner *et al*., 2012), emphasizing the contribution of PGC1α to maintaining insulin sensitivity. There was a decrease in the expression of PGC1α in SQ fat of df/df -VFR mice. Even though there was no significant change in the expression of PPARγ in this fat depot upon VFR, the decrease in PGC1α transcript levels could potentially lead to a decrease in insulin sensitivity in df/df-VFR mice. The expression of IR and IRS-1 at the mRNA level was higher in df/df mice compared with N mice in the sham-operated groups, which could contribute to the enhanced insulin sensitivity of these mutant dwarfs.

To understand how VF in df/df mice contributes to enhanced insulin sensitivity of these mutants, we analyzed the expression of insulin signaling genes and adipokine levels in EF of df/df and N mice. The expression of IR, IRS-1, and PGC1α was increased in the VF depot of df/df mice compared with the controls. Moreover, mRNA levels of pro-inflammatory TNFα were significantly decreased in EF of df/df mice. Moreover, the levels of IL-6 protein, another pro-inflammatory adipokine, were significantly lower in EF depot of these mutants. Protein levels of adiponectin, which has anti-inflammatory and antidiabetic properties, were higher in this VF pad compared with SQ fat of df/df mice. Concomitant with decreased expression of IL-6 and increased expression of adiponectin proteins in EF of df/df mice, these mutant dwarfs also had low levels of IL-6 and high levels of adiponectin in circulation. However, VFR did not significantly alter plasma adiponectin levels in either group of mice. One possible explanation of this observation is the fact that the removal of EF and PR fat depots causes an increase in the secretion of adiponectin by intact SQ fat depots as a compensatory mechanism. Thus, the present findings may explain the contribution of EF to improved insulin signaling in df/df mice. There were no significant differences in the protein expression of adiponectin, as measured by ELISA, between the major VF depots (EF and PR fat) or SQ fat between the two groups of mice. Also, EF and PR fat depots of N mice had a higher protein level of adiponectin as compared to SQ fat. Several studies support the idea that VF produces adiponectin and thus determines the level of adiponectin in circulation (Halleux *et al*., 2001; Motoshima *et al*., 2002; Cnop *et al*., 2003). In the current study, we used healthy, nonobese mice for determining physiological adiponectin levels in different fat pads rather than dysregulation in the production of adiponectin associated with genetic or dietary induction of obesity. Under obese conditions, the different fat depots would have a differential secretory profile, with VF releasing more pro-inflammatory adipokines and less adiponectin.

Even though df/df mice share several characteristics with GHRKO mice, these two groups of mutant mice exhibit traits exclusive to their genotypes. Most importantly, cells of the df/df mouse do express the GHR, but due to the *prop1* mutation, somatotrophs do not produce GH. Conversely, cells of the GHRKO mouse are characterized by the absence of functional GHRs, but there is pituitary production of GH. Thus, a state of GH deficiency or GH resistance can be observed in the df/df and GHRKO mice, respectively.

**Figure 5:**
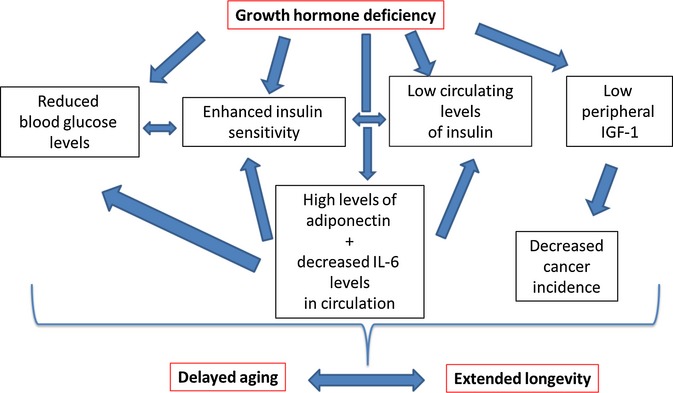
Proposed mechanisms of extended longevity in df/df mice. GH-deficient df/df mice are very insulin sensitive with low levels of glucose and insulin in circulation. These mutant mice also have high levels of adiponectin and decreased levels of IL-6 in circulation. Due to GH deficiency, df/df mice have very low IGF-1 in circulation and, thus, a decreased incidence of cancer. All these findings taken together could lead to a delayed rate of aging and, thus, an extension of longevity of df/df mice.

Summing up, our present results suggest that EF (one of the major VF depots in mice), developed in the absence of GH signals, has decreased mRNA levels of proinflammatory cytokines – TNFα and IL-6 with parallel increase in the levels of anti-inflammatory adiponectin. These alterations may contribute to increased whole-body insulin sensitivity and enhancement of insulin action in skeletal muscle of these long-living mutant mice. However, the expression of insulin signaling genes by measurements of the mRNA levels should be followed in the future by determinations of the levels of the corresponding proteins and phosphoproteins. These analyses were not carried out in the present study due to limited amount of tissues that could be dissected from these diminutive animals. However, based on presented results, we can conclude that at the very least, VF in df/df mice does not negatively affect insulin signaling and is not detrimental to the overall health of these animals. These new data obtained from GH-deficient Ames dwarf mice as well as previously published findings in GH-resistant Laron dwarf mice suggest that the impact of reduced somatotropic axis activity on insulin signaling and inflammation importantly contributes to delayed aging and extended healthspan and longevity of these remarkably long-lived mutants (Fig. 5).

## Materials and Methods

### Experimental animals

Breeding was carried out as described previously (Bartke *et al*., 2001). Ames dwarf and control male mice used in this study were developed by crossing normal heterozygous (df+/−) female with homozygous Ames dwarf male mice; thus, 50% of the progeny were mutant. The *prop1* mutation was maintained on a heterogeneous background. All mice were maintained in temperature-controlled conditions (20–22 °C) under 12-h light/12-h dark cycles. They had free access to drinking water and standard laboratory chow diet. Ames dwarf mice were weaned at 41 days, while N mice were weaned at the usual 21 days. Animals were provided *ad libitum* access to food with nutritionally balanced diet (Rodent Laboratory Chow 5001; 23.4% protein, 4.5% fat, 5.8% crude fiber; LabDiet PMI Feeds, Inc., St. Louis, MO, USA).

### Visceral fat removal

Ames dwarf and N male mice, 3–6 months old, were categorized into two groups: visceral fat removal (VFR) and sham-operated, respectively. All surgical procedures were carried out under ketamine/xylazine anesthesia following a previously established protocol (Masternak *et al*., 2012). Mice in the sham-operated group had their VF mobilized, but not removed.

### Animals for analysis of fat adiponectin and serum IL-6 levels

Animals used for analyzing the levels of adiponectin in different fat pads and IL-6 in serum were about 1-year-old males. After an overnight fast, df/df (*n* = 10) and N (*n* = 10) mice were anesthetized with isofluorane. Blood was collected by cardiac puncture. Mice were sacrificed by cervical dislocation. Different fat pads (EF, PR, and SQ) were collected and snap-frozen in liquid nitrogen. Proteins were extracted from these fat depots before storing the tissues at −80 °C.

### Analysis of glucose tolerance and whole-body insulin sensitivity

Approximately a month after surgery, mice (*n* = 16–20) were subjected to a glucose tolerance test (GTT) to determine their ability to clear blood glucose. After overnight fasting, body weights were determined and the tail was snipped to collect blood samples. A baseline blood glucose measurement (0 min) was made using a standard glucometer. Then, the mice were injected intraperitoneally with glucose (2 g glucose per kg body weight), and blood glucose levels were measured at defined time points (15 min, 30 min, 45 min, 60 min, and 120 min). Following GTT, 100 animals from each group were randomly selected to perform an insulin tolerance test (ITT) to determine sensitivity to injected insulin at the whole-body level. For this experiment, mice were not subjected to standard 4-h fasting before the test; however, the food was removed from the cages in the morning when the animals were weighed and then the baseline blood glucose (0 min) was analyzed. This method was modified based on our previous work with insulin-sensitive df/df and GHRKO mice (26) when we observed that subjecting these mice to 4-h fasting before ITT caused glucose levels dropping below 20 mg dL^−1^. Following our observations, we had to start to perform ITT testing with food removal during body weights collection initially before the insulin injection which eliminated previous hypoglycemia problems. After initial blood glucose collection, the mice were intraperitoneally injected with insulin (1 IU insulin per kg body weight). Blood glucose levels were checked at specific time points (15 min, 30 min, 45 min, and 60 min). Animals not responding to the injected insulin as indicated by an increase in glucose level after insulin injection were excluded from final analysis as nonresponders which resulted in *n* = 6–9 animals per group as a final count for ITT.

### Insulin stimulation and tissue collection

Three months after surgery, mice were fasted overnight and then anesthetized with ketamine/xylazine. Blood was collected by cardiac puncture. In each group of mice, animals (approximately 50%) were injected with either a high dose of insulin (10 IU insulin per kg body weight) or saline (control) via the inferior vena cava to stimulate insulin signaling in target organs. Exactly 2 min after injection, mice were sacrificed by cervical dislocation, and tissues were collected and snap-frozen in liquid nitrogen and stored at −80 °C till further analysis. At the time of tissue collection, no visible regrowth of fat was seen in the animals that had undergone VFR.

### RNA extraction from frozen tissue

RNA was extracted from approximately 50 mg of frozen skeletal muscle and adipose tissues using the miRNeasy mini kit (Qiagen, Valencia, CA, USA) according to the manufacturer’s protocol. Samples were homogenized using zirconium oxide beads (1.0 mm for skeletal muscle and 0.5 mm for adipose tissue) using the Bullet Blender Homogenizer BBX24 (Next Advance, Averill Park, NY, USA) (Skeletal muscle: speed 10, 3 min; adipose tissue: speed 8, 3 min). RNA extraction from adipose tissue involved the additional step of centrifuging the samples at 12 000 × *g* for 10 min at 4 °C, after which the upper lipid layer was excluded and only the infranatant was transferred to a new tube. Pure RNA was eluted using 50 μL and 30 μL nuclease-free water (provided in the kit) for skeletal muscle and adipose tissue samples, respectively. The concentration and purity of the eluted RNA samples were determined using the Take3 plate and the Epoch plate reader (BioTek, Winooski, VT, USA). RNA samples with A260/A280 < 1.9 were re-extracted. Extracted RNA samples were stored at −80 °C until further use.

### cDNA synthesis

Complementary DNA was synthesized from extracted RNA samples using iScript™ cDNA Synthesis Kit (Bio-Rad, Hercules, CA, USA) (Skeletal muscle and epididymal fat) or EasyScript Plus™ cDNA Synthesis Kit (Applied Biological Materials, Richmond, BC, Canada) (Subcutaneous fat) according to the manufacturer’s protocol. One microgram of RNA was used per reaction.

### Quantitative real-time polymerase chain reaction (qPCR)

Relative gene expression was analyzed by qPCR using Fast SYBR® Green Master Mix (Applied Biosystems, Foster City, CA, USA) on 7900 HT fast real-time PCR system (Applied Biosystems) with the following run conditions: enzyme activation at 95 °C for 20 s, denaturation at 95 °C for 1 s, and annealing/extension at 62 °C for 20 s. Additionally, a melt curve was included for each run. Forward and backward primer sequences used are as in Table 1. Results were analyzed as detailed previously (Masternak *et al*., 2005).

**Table 1:** List of primers used for qPCR

Gene	Primer sequences (forward and reverse)
B2M	F: 5′-AAGTATACTCACGCCACCCA-3′
	R: 5′-CAG CGC TAT GTA TCA GTC TC-3′
IR	F: 5′-GTTCTTTCCTGCGTGCATTTCCCA-3′
	R: 5′-ATCAGGGTGGCCAGTGTGTCTTTA-3′
IRS-1	F: 5′- AGCCCAAAAGCCCAGGAGAATA-3′
	R: 5′-TTCCGAGCCAGTCTCTTCTCTA-3′
IRS-2	F: 5′-AGTAAACGGAGGTGGCTACA-3′
	R: 5′-AAGCTGAGAAGTCAAGGT-3′
PI3K	F: 5′-TAGCTGCATTGGAGCTCCTT-3′
	R: 5′-TACGAACTGTGGGAGCAGAT-3′
Akt2	F: 5-GAGGACCTTCCATGTAGACT-3′
	R: 5′-CTCAGATGTGGAAGAGTGAC-3′
GLUT4	F: 5′-ATTGGCATTCTGGTTGCCCA-3′
	R: 5′-GGTTCCGGATGATGTAGAGGTA-3′
PPARγ	F: 5′-GTCAGTACTGTCGGTTTCAG-3′
	R: 5′-CAGATCAGCAGACTCTGGGT-3′
Pgc1α	F: 5′-TACGCAGGTCGAACGAAACT-3′
	R: 5′-TGCTCTTGGTGGAAGCA-3′
TNFα	F: 5′-TAGCAAACCACCAAGTGGAG-3′
	R: 5′-AACCTGGGAGTAGACAAGGT-3′
IGF-1	F: 5′-CTGAGCTGGTGGATGCTCTT-3′
	R: 5′-CACTCATCCACACCTGT-3′

### Protein extraction from frozen tissue

Total proteins were extracted from skeletal muscle and adipose tissues using T-PER Tissue Protein Extraction Reagent (with proteases and phosphatases inhibitors) (Thermo Scientific, Waltham, MA, USA). Samples were homogenized in the extraction buffer using the Bullet Blender homogenizer, as mentioned earlier. After centrifugation at 12 000 × *g* for 15 min at 4 °C, the layer below the fat was collected into a new tube and centrifuged again. The relatively fat-free protein extract was then quantified using the BCA assay (Thermo Scientific). For the quantification assay, protein samples were diluted 1:10 with T-per.

### Analysis of insulin and adiponectin levels

Ultra sensitive mouse insulin ELISA kit (Crystal Chem, Downers Grove, IL, USA) was used for the analysis of insulin in plasma. Mouse adiponectin ELISA kits (Invitrogen, Grand Island, NY, USA) were used to analyze the levels of adiponectin in plasma and different fat pads. All procedures were performed according to the manufacturer’s protocol. For analyzing adiponectin levels in fat tissue, extracted proteins were first adjusted to a concentration of 0.5 μg μL^−1^ in a total volume of 60 μL in the same extraction buffer (T-PER) and then diluted to a concentration of 0.0025 μg μL^−1^ using the diluent buffer (1X) from the kit. The protocol provided in the kit was then followed.

### Bio-Plex Pro™ Assay for IL-6 levels in serum

Interleukin-6 levels in serum of df/df and N mice were detected using the Bio-plex assay (Bio-Rad) according to the provided protocol.

### Statistical analysis

Analyses were performed by two-way ANOVA test and Student’s t-test within groups when warranted. All statistics and graphs were conducted using Prism 5 (GraphPad Software, San Diego, CA, USA). Alpha is set to 0.05. Values are reported as mean ± standard error of the mean (SEM) throughout the figures and text.

## Supporting Information

**Fig. S1** Effect of VFR on body weight of df/df and N mice. Different letters represent statistical significance (*P* < 0.05). **Fig. S2** Visceral fat content (absolute and percentage of body weight) of N and df/df mice. Different letters represent statistical significance (*P* < 0.05).
