# Supplementary material for: The contribution of visceral fat to improved insulin signaling in Ames dwarf mice
Source: Aging Cell. 2014 Feb 12;13(3):497–506. doi: 10.1111/acel.12201 (PMC4032618; doi:10.1111/acel.12201)
Supplement: Supplementary file 1 — Fig. S1 Effect of VFR on body weight of df/df and N mice. Different letters represent statistical significance (P < 0.05). Fig. S2 Visceral fat content (absolute and percentage of body weight) of N and df/df mice. Different letters represent statistical significance (P < 0.05). [file acel0013-0497-sd1.docx]

Supplement 1

Supplement 2
